# Maternal depression and offspring mental health at age 5: MINA-Brazil cohort study

**DOI:** 10.11606/s1518-8787.2023057005560

**Published:** 2024-02-01

**Authors:** Alicia Matijasevich, Alexandre Faisal-Cury, Isabel Giacomini, Julia de Souza Rodrigues, Marcia C. Castro, Marly A. Cardoso

**Affiliations:** I Universidade de São Paulo Faculdade de Medicina Departamento de Medicina Preventiva São Paulo SP Brasil Universidade de São Paulo. Faculdade de Medicina. Departamento de Medicina Preventiva. São Paulo, SP, Brasil; II Universidade de São Paulo Faculdade de Saúde Pública Programa de Pós-Graduação em Nutrição em Saúde Pública São Paulo SP Brasil Universidade de São Paulo. Faculdade de Saúde Pública. Programa de Pós-Graduação em Nutrição em Saúde Pública. São Paulo, SP, Brasil; III Universidade de São Paulo Faculdade de Medicina Programa de Pós-Graduação em Saúde Coletiva São Paulo SP Brasil Universidade de São Paulo. Faculdade de Medicina. Programa de Pós-Graduação em Saúde Coletiva. São Paulo, SP, Brasil; IV Harvard T.H. Chan School of Public Health Department of Global Health and Population Boston MA United States of America Harvard T.H. Chan School of Public Health. Department of Global Health and Population. Boston, MA, United States of America; V Universidade de São Paulo Faculdade de Saúde Pública Departamento de Nutrição São Paulo SP Brasil Universidade de São Paulo. Faculdade de Saúde Pública. Departamento de Nutrição. São Paulo, SP, Brasil

**Keywords:** Mental Health, Maternal Depression, Cohort Studies

## Abstract

**OBJECTIVE:**

To identify longitudinal patterns of maternal depression between three months and five years after child's birth, to examine predictor variables for these trajectories, and to evaluate whether distinct depression trajectories predict offspring mental health problems at age 5 years.

**METHODS:**

We used data from the Maternal and Child Health and Nutrition in Acre (MINA-Brazil) study, a population-based birth cohort in the Western Brazilian Amazon. Maternal depressive symptoms were assessed with the Edinburgh Postnatal Depression Scale (EPDS) at 3 and 6–8 months, and 1 and 2 years after delivery. Mental health problems in 5-year-old children were evaluated with the Strengths and Difficulties Questionnaire (SDQ) reported by parents. Trajectories of maternal depression were calculated using a group-based modelling approach.

**RESULTS:**

We identified four trajectories of maternal depressive symptoms: "low" (67.1%), "increasing" (11.5%), "decreasing" (17.4%), and "high-chronic" (4.0%). Women in the "high/chronic" trajectory were the poorest, least educated, and oldest compared with women in the other trajectory groups. Also, they were more frequently multiparous and reported smoking and having attended fewer prenatal consultations during pregnancy. In the adjusted analyses, the odds ratio of any SDQ disorder was 3.23 (95%CI: 2.00–5.22) and 2.87 (95%CI: 1.09–7.57) times higher among children of mothers belonging to the "increasing" and "high-chronic" trajectory groups, respectively, compared with those of mothers in the "low" depressive symptoms group. These differences were not explained by maternal and child characteristics included in multivariate analyses.

**CONCLUSIONS:**

We identified poorer mental health outcomes for children of mothers assigned to the "chronic/severe" and "increasing" depressive symptoms trajectories. Prevention and treatment initiatives to avoid the adverse short, medium, and long-term effects of maternal depression on offspring development should focus on women belonging to these groups.

## INTRODUCTION

Depression is one of the most important public health problems worldwide, affecting mainly women^1^. Perinatal depression, a global public health issue, and more frequent among women from low- and middle-income countries, encompasses major and minor depressive episodes that occur either during pregnancy or within 12 months after delivery^2^. Postpartum depression (PPD) may occur at any time during the first year after delivery and continue for several years. A recent review of the literature with 565 studies from 80 different countries or regions found a prevalence of PPD of 17.2% (95%CI: 16.0–18.5), with higher figures among middle- and low-income countries^3^.

Maternal depression frequently occurs in a context of socioeconomic problems, stressful live events, single motherhood, marital difficulties, and little social support. Among vulnerable women, complications during pregnancy and childbirth as well as child health problems can also trigger depressive symptoms^4^. Although severe cases of PPD can be easily detected, moderate cases are often confused with tiredness or sleep disturbances, or even go completely unnoticed by family members and health professionals^5^. Most depressive symptoms after childbirth are self-limiting, disappearing within a few months of their onset^6^. However, maternal depressive symptoms can become chronic or recurrent considering that the previous history of depression and antenatal depression are important risk factors for PPD^7^.

In addition to the impairments in woman's quality of life and the negative impact on familial relationships, maternal depression has also negative consequences on child development. Exposure to PDD leads to negative outcomes in multiple domains of child health, ranging from higher rates of prematurity and low birthweight, malnutrition and impairment in language, cognitive and motor development^8^. The PDD is also associated with an increased risk of offspring behavioural difficulties and persistent internalizing and externalizing disorders with effects that go far beyond childhood^9^.

Clinical evidence has demonstrated heterogeneity in the patterns and predictors of maternal depressive symptomatology over time. A recent systematic review identified nine studies, mostly from high-income countries, that modelled trajectories of maternal depression and studied the impact of these trajectories on child psychiatric disorders^10^. This study, conducted in the Amazonian region, has three main objectives: 1) to identify trajectories of maternal depression between three months and 5 years after the child's birth, 2) to examine predictor variables for these trajectories, and 3) to evaluate whether maternal depression trajectories predict offspring mental health problems at 5 years of age.

## METHODS

### Participants

The Maternal and Child Health and Nutrition Study in Acre (MINA-Brazil) is a population-based birth cohort of children born from July 2015 to June 2016 in the city of Cruzeiro do Sul, state of Acre, Brazil. The municipality of Cruzeiro do Sul is in the North region of the country, in the Western Amazon, with an estimated population of 88,376 people in 2019. In 2010, the Human Development Index of Cruzeiro do Sul was 0.664, compared with 0.727 in Brazil. The fieldwork team visited daily the only maternity hospital in the city and invited all women who gave birth in the hospital to participate in the study. Mothers were interviewed soon after delivery using a structured questionnaire. Information was obtained on demographic, socioeconomic, behavioural, and biological characteristics, reproductive history, and health care utilization. Characteristics of their newborns, such as gender, gestational age, weight, and length at birth, were obtained from hospital records. The baseline follow-up consisted of 1,246 children born to mothers residing in the urban area. Only single births were included in the study. Mothers and children residing in the remote rural area of the city were not eligible for follow-up. After birth, the 3-month follow-up visit was carried out by telephone interview. Assessments at 6–8 months, 1, 2, and 5 years after delivery were scheduled by telephone and carried out in a basic health unit in the city. During the follow-ups, information about several characteristics of the family, mother, and child were collected. In case of non-attendance, up to three rescheduling attempts were made. More details of the methodology were previously described^11^.

### Measures

**Maternal depressive symptoms:** repeated assessments of maternal depressive symptoms at 3 and 6–8 months, and 1 and 2 years after delivery were made using the Edinburgh Postnatal Depression Scale (EPDS)^12^. The EPDS is a self-administered, 10-item scale; each item has four possible responses ranging from 0 to 3, with a total minimum score of 0 and maximum of 30. The scale indicates the intensity of depressive symptoms over the preceding seven days. We used a Brazilian version of the questionnaire, validated previously in Brazil^13^. The validation study showed that a cut-off score of ≥ 10 identified women at risk of minor depression with 82.6% (75.3–89.9%) sensitivity and 65.4% (59.8–71.1%) specificity. The EPDS has also been validated to be used in the adult population outside of the postpartum period^14^. In the MINA-Brazil study, maternal scores on the EPDS correlated moderately over time (from 0.41 to 0.67).

**Child's mental health assessment:** at the five-year follow-up visit, mothers were interviewed by trained fieldworkers using the Strengths and Difficulties Questionnaire (SDQ): a brief behavioural screening questionnaire for 2–17-year-olds. The SDQ was validated for use in Brazil by Fleitlich-Bilyk and Goodman^15^. The instrument consists of 25 questions divided into five subscales of five items each: emotional symptoms, conduct problems, hyperactivity/inattention, peer relationship problems, and prosocial behaviour. Each question is scored on a three-point scale (not true = 0; somewhat true = 1; certainly true = 2). We used total SDQ scores and those of the emotional, conduct, attentional/hyperactivity, and peer problems subscales. The total difficulty score (any SDQ difficulty) was calculated by adding the results of the subscales (except prosocial behaviour). The resulting score ranged from 0 to 40 and could be stratified into three categories: normal (0–13), borderline (14–16), and abnormal (17–40). The subscale scores ranged from 0 to 10 and were also categorized as normal, borderline, or abnormal, according to the suggested cut-off points available on the SDQ website^16^. We used a dichotomous classification of SDQ scores, whereby subjects in the borderline and abnormal categories were compared with those in the normal group.

**Covariates:** information on maternal and child variables was collected in the perinatal interview. A wealth index, estimated by principal component analysis, was based on domestic possessions of consumer goods and characteristics of infrastructure and housing. It was categorized into quintiles, with the 1^st^ and the 5^th^ quintiles the poorest and the wealthiest groups, respectively. Maternal schooling at the time of delivery was recorded as complete school years and categorized into ≤ 9, 10–12, and > 12 years of formal education. Maternal age was recorded in complete years and categorized into < 19, 20–35, and > 35 years. Women who were single, widowed, divorced, or lived without a partner were classified as single mothers. Maternal skin colour was self-reported and categorized as white or Black/mixed race. Maternal smoking behaviour during pregnancy was assessed retrospectively at birth by self-report. Regular smokers were defined as those women who smoked at least one cigarette daily in any trimester of pregnancy. Any amount of alcohol intake during any trimester of pregnancy was considered as alcohol consumption during pregnancy. Women were asked if they planned their pregnancy (yes/no). Parity was defined as the number of previous viable pregnancies and categorized as 1 (primiparity) and ≥ 2. Number of prenatal care visits was categorized as < 6 and ≥ 6. The type of delivery was classified as vaginal or by caesarean section.

Child variables were recorded at birth. Birthweight was measured by hospital staff. Low birthweight was defined as less than 2,500g. Gestational age was based on the last menstrual period, ultrasound, and clinical maturity of the newborn. Births before the 37^th^ week of pregnancy were classified as preterm.

### Analysis Plan

First, trajectories of depressive symptoms, based on EPDS scores from three-months until the five-year-follow-up, were created by applying a semiparametric, group-based modelling approach, a specialized form of finite mixture modelling^17^. The method is designed to identify rather than assume groups or clusters of individuals following similar developmental trajectories. A polynomial function is used to model the relationship between an attribute (i.e. maternal depression symptomatology) and age or time^17,18^. The models were estimated with the Stata procedure "traj"^19^. We included 1,141 women with valid EPDS data from at least one follow-up visit. The proportion of women that completed the EPDS in all follow-ups was 29.4%. Overall, 71.4% completed the EPDS at least three times, 16.2% completed the EPDS two times and 12.4% completed the EPDS only one time. Individuals with missing information were not excluded from the model due to group-based trajectory modelling handling missing data using maximum likelihood estimation^17^. However, women who did not complete the EPDS (n = 105) were excluded from the analyses.

To estimate maternal depression trajectories, a censored normal (CNORM) model was fitted to the data. The number and shape of trajectories were chosen based on the best fit of the model maximum Bayesian information criteria (BIC), the interpretability of the trajectories obtained, and the posterior probability scores for each trajectory group (i.e., the individual's probability of belonging to each of the trajectory groups). According to Nagin^17^, an average probability score should be higher than 0.70 for all groups.

In a second stage of analyses, we examined the contribution of predictor variables (maternal and child's characteristics) on the maternal depression trajectory group. Since variables were categorical, we used the chi-square test to compare the groups on maternal and child characteristics.

In the third stage, we evaluated whether maternal depression trajectory group membership predicted child's mental health problems at 5 years old. We had valid information on SDQ for 694 children. Multivariate logistic regression analysis was used to estimate the association between any SDQ difficulty, emotional symptoms, conduct problems, hyperactivity/inattention, and peer relationship problems disorders (binary outcomes) and trajectories of maternal depressive symptoms (exposure) adjusting for potential confounding factors in separate models. Potential confounding variables were included in the adjusted analysis using a backward strategy selection. Each outcome had three models: unadjusted results (model 1), results adjusted for maternal characteristics (model 2), and results adjusted for model 2 variables plus child characteristics (model 3). If the significance level was below 0.20^20^, the variable remained in the model as a potential confounder for the next level. Interaction terms between maternal trajectories of depressive symptoms and child gender were tested but not introduced into the model, since they did not reach statistical significance. All analyses were performed with Stata software version 17.0 (StataCorp LP, College Station, Tex).

### Ethics

The study protocol and all follow-ups of the MINA-Brazil cohort study were approved by the Ethics Committee of the Universidade de São Paulo. A signed informed consent form was used in each follow-up, after informing mothers of the study objectives. Data confidentiality and voluntary participation were guaranteed throughout, as was the option to withdraw from the study at any time, without offering a reason. Those mothers identified with serious mental health problems were referred to the appropriate local healthcare facilities.

## RESULTS

### Attrition Analyses

Of the 1,246 participants, 1,141 (91.6%) women had valid EPDS data from at least one follow-up, and, of those, 694 answered the SDQ questionnaire (56% of the original cohort). Among members not included either in the analyses of maternal depression trajectories or in the analyses of the association between maternal depression trajectories and offspring mental health problems, women belonging to the poorest families, with the lowest education, adolescent mothers, those who consumed alcohol during pregnancy, and who had the fewest number of prenatal care visits were overrepresented (Table 1).

**Table 1:** Baseline characteristics of mothers and children included and not included in this study.

Variables		Analyses of maternal depression trajectories			Analyses of the association between maternal depression trajectories and offspring mental health problems		
		Included	Not included	p-value	Included	Not included	p-value^a^
		n = 1,141	n = 105		n = 694	n = 552	
Wealth index (quintiles) (%)				0.005			< 0.001
	1^st^ (poorest)	19.0	29.3		13.0	28.6	
	2^nd^	19.4	28.3		16.4	24.7	
	3^er^	20.4	14.1		22.0	17.2	
	4^th^	20.3	18.5		23.2	16.3	
	5^th^ (wealthiest)	20.9	9.8		25.4	13.2	
Schooling (years) (%)				0.004			<0.001
	≤ 9	34.2	51.1		26.2	47.3	
	10–12	47.8	38.0		51.2	41.8	
	> 12	18.0	10.9		22.6	10.9	
Maternal age (years) (%)				0.020			< 0.001
	< 19	17.9	28.3		16.0	22.1	
	20–35	72.4	66.7		72.1	71.8	
	> 35	9.7	5.0		11.9	6.1	
Single mother (%)				0.495			0.329
	No	77.3	80.4		78.6	76.3	
	Yes	22.7	19.6		21.4	23.7	
Maternal skin colour (%)				0.009			0.799
	White	12.0	18.5		12.4	12.6	
	Black/mixed race	82.1	69.6		81.6	80.5	
	Yellow/Indigenous	5.9	11.9		6.0	6.9	
Smoking during pregnancy (%)				0.187			0.028
	No	95.4	92.4		96.4	93.7	
	Yes	4.6	7.6		3.6	6.3	
Alcohol consumption during pregnancy (%)				0.001			0.002
	No	83.0	69.6		85.0	78.0	
	Yes	17.0	30.4		15.0	22.0	
Planned pregnancy (%)				0.878			0.182
	No	60.0	60.9		58.4	62.3	
	Yes	40.0	39.1		41.6	37.7	
Parity (%)				0.578			0.613
	1 (Primiparity)	41.6	44.6		42.4	41.0	
	≥ 2	58.4	55.4		57.6	59.0	
Number of prenatal care visits (%)				< 0.001			< 0.001
	< 6	22.0	43.9		16.3	33.1	
	≥ 6	78.0	56.1		83.7	66.9	
Type of delivery (%)				0.582			0.041
	Vaginal	53.7	56.6		51.3	57.2	
	Caesarean section	46.3	43.4		48.7	42.8	
Child sex (%)				0.527			0.337
	Male	49.8	53.5		50.7	48.0	
	Female	50.2	46.5		49.3	52.0	
Low birthweight (< 2,500g) (%)				0.213			0.382
	No	93.2	89.9		93.5	92.3	
	Yes	6.8	10.1		6.5	7.7	
Preterm birth (< 37 weeks) (%)				0.013			0.280
	No	92.1	84.8		92.3	90.6	
	Yes	7.9	15.2		7.7	9.4	

a Chi-square test.

### Sample Description

Approximately half of the women had 10–12 years of formal education, 70% were aged between 20 and 35 years, one out of five were single mothers, most of them self-classified as being Black or Mixed race, did not smoke or consume alcohol during pregnancy, had 6 or more antenatal care visits, and around 40% were primiparous and did not plan the pregnancy. The most frequent route of delivery was vaginal and approximately 7% and 8% of the children had low birthweight and were preterm at birth, respectively.

### Identification of Trajectories

We modelled maternal EPDS scores trajectories from the time the children were 3 months to 5 years of age. Analyses were conducted specifying three-, four-, five-, and six-group models. The BIC improved as more groups were added, however, the improvement observed from the four-group to the five- and six-group model was very low and the four-group emerged as the best fitting and more parsimonious model. Regarding parameter estimates for the four-group model, Table 2 shows that the constant term differed from zero for all four groups. Two trajectories were best represented by a cubic term, one trajectory was linear and the other one was quadratic (Figure). Group 1 (named "low", n = 765, 67.1%) had EPDS scores below 5 across all time points, suggesting low depressive symptomatology. Group 2 (named "increasing", n = 131, 11.5%) showed EPDS scores below 10 in the first 24 months postpartum with a sharp increase between the 24-month and 5-year follow-up. Group 3 (named "decreasing", n = 199, 17.4%) showed a consistent decrease in depressive symptoms during the study period. Finally, Group 4 (named "high/chronic", n = 46, 4.0%) represents women that had very high EPDS scores all over the study period. For all four groups, the average posterior probability (APP) was above the lower recommended threshold for assignment, 0.7 (APP of 0.89, 0.74, 0.73, and 0.87 for Group 1 to Group 4, respectively).

**Figure:**
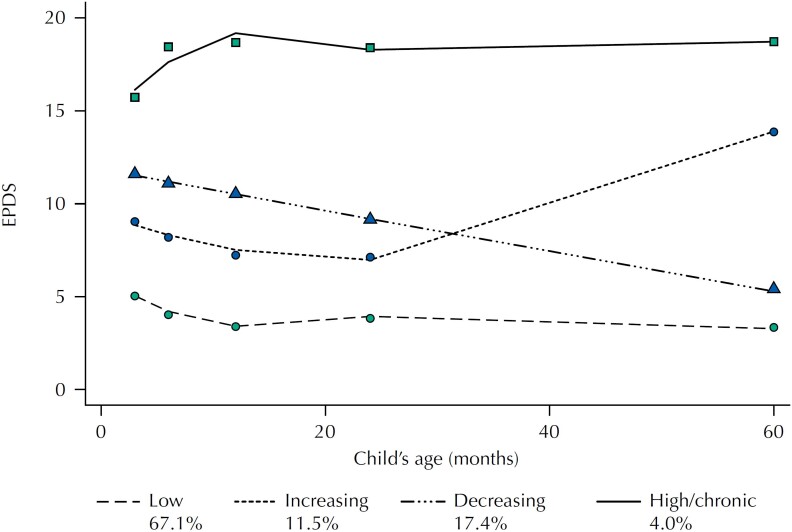
Trajectories of maternal depression symptoms measured by the Edinburgh Postpartum Depression Score (EPDS) by child's age.

**Table 2:** Descriptive and average posterior probabilities (APP) for the trajectories (n = 1,141).

Trajectories of depressive symptoms	n (%)	APP (SD)	Parameters	Parameter estimates		
				B	(SE)	p-value
1 "Low" depressive symptoms	765 (67.1)	0.89 (0.15)	Intercept	6.205	(0.312)	< 0.001
			Linear	−0.503	(0.071)	< 0.001
			Quadratic	0.022	(0.003)	< 0.001
			Cubic	−0.000	(0.000)	< 0.001
2 "Increasing" depressive symptoms	131 (11.5)	0.74 (0.22)	Intercept	9.474	(0.551)	< 0.001
			Linear	−0.225	(0.048)	< 0.001
			Quadratic	0.005	(0.001)	< 0.001
3 "Decreasing" depressive symptoms	199 (17.4)	0.73 (0.19)	Intercept	11.861	(0.428)	< 0.001
			Linear	−0.112	(0.015)	< 0.001
4 "High/chronic" depressive symptoms	46 (4.0)	0.87 (0.17)	Intercept	14.081	−1.336	< 0.001
			Linear	0.784	(0.309)	0.011
			Quadratic	−0.034	(0.015)	0.027
			Cubic	0.000	(0.000)	0.037

### Factors Associated with Maternal Depression Trajectory Membership

Women in the "high/chronic" depression trajectory group belonged to the poorest families and were more frequently the least educated, older, and multiparous. They also reported smoking more frequently during pregnancy and having attended fewer prenatal consultations than women in the other trajectory groups. Regarding child characteristics, low birthweight was higher among offspring of mothers belonging to the high/chronic trajectory than among those of mothers belonging to the other groups (Table 3).

**Table 3:** Maternal and child characteristics according to maternal depression trajectory groups.

Variables^a^		Maternal depression trajectory groups				p-value^b^
		Group 1 "Low"	Group 2 "Increasing"	Group 3 "Decreasing"	Group 4 "High/chronic"	
		n (%)	n (%)	n (%)	n (%)	
Wealth index (quintiles)						< 0.001
	1^st^ (poorest)	120 (16.3)	24 (19.0)	48 (24.6)	17 (40.5)	
	2^nd^ to 5^th^	616 (83.7)	102 (81.0)	147 (75.4)	25 (59.5)	
Schooling (years)						< 0.001
	≤ 9	218 (29.7)	49 (38.9)	83 (42.6)	25 (59.6)	
	10–12	353 (48.0)	62 (49.2)	96 (49.2)	14 (33.3)	
	> 12	164 (22.3)	15 (11.9)	16 (8.2)	3 (7.1)	
Maternal age (years)						0.015
	< 19	139 (18.4)	21 (16.3)	39 (19.7)	2 (4.6)	
	20–35	551 (73.1)	91 (70.5)	141 (71.2)	32 (72.7)	
	> 35	64 (8.5)	17 (13.2)	18 (9.1)	10 (22.7)	
Single mother						0.380
	No	563 (76.5)	95 (75.4)	160 (82.0)	32 (76.2)	
	Yes	173 (23.5)	31 (24.6)	35 (18.0)	10 (23.8)	
Maternal skin colour						0.644
	White	86 (11.7)	16 (12.7)	21 (10.8)	9 (21.4)	
	Black/Mixed race	608 (82.6)	102 (81.0)	161 (82.5)	31 (73.8)	
	Yellow/Indigenous	42 (5.7)	8 (6.3)	13 (6.7)	2 (4.8)	
Smoking during pregnancy						< 0.001
	No	713 (96.9)	119 (94.4)	183 (93.8)	34 (80.9)	
	Yes	23 (3.1)	7 (5.6)	12 (6.2)	9 (19.1)	
Alcohol consumption during pregnancy						0.255
	No	617 (83.8)	107 (84.9)	157 (80.5)	31 (73.8)	
	Yes	119 (16.2)	19 (15.1)	38 (19.5)	11 (26.2)	
Planned pregnancy						0.086
	No	428 (58.1)	74 (58.7)	133 (68.2)	25 (59.5)	
	Yes	308 (41.9)	52 (41.3)	62 (31.8)	17 (40.5)	
Parity						< 0.001
	1 (Primiparity)	348 (47.3)	38 (30.2)	64 (32.8)	7 (16.7)	
	≥ 2	388 (52.7)	88 (69.8)	131 (67.2)	35 (83.3)	
Number of prenatal care visits						< 0.001
	< 6	146 (19.5)	26 (20.3)	54 (27.8)	19 (43.2)	
	≥ 6	604 (80.5)	102 (79.7)	140 (72.2)	25 (56.8)	
Type of delivery						0.664
	Vaginal	397 (52.6)	70 (54.3)	114 (57.6)	23 (52.3)	
	Caesarean section	357 (47.4)	59 (45.7)	84 (42.4)	21 (47.7)	
Child sex						0.838
	Male	372 (49.3)	69 (53.5)	98 (49.5)	21 (47.7)	
	Female	382 (50.7)	60 (46.5)	100 (50.5)	23 (52.3)	
Low birthweight (< 2,500g)						0.003
	No	706 (93.8)	120 (93.0)	187 (94.4)	35 (79.5)	
	Yes	47 (6.2)	9 (7.0)	11 (5.6)	9 (20.5)	
Preterm birth (< 37 weeks)						0.141
	No	686 (91.5)	120 (93.0)	186 (95.4)	38 (86.4)	
	Yes	64 (8.5)	9 (7.0)	9 (4.6)	6 (13.6)	

a Totals differ due to missing values.

b Chi-square test.

### Children's Mental Health Problems at 5 Years as a Function of Maternal Depression Trajectory Membership

Regarding children's mental health problems, 33.1% (n = 230) had some SDQ difficulty at five years. Emotional and hyperactivity/inattention symptoms were 39.9% (n = 277) and 33.1% (n = 230), respectively. The most frequent disorders reported by the mothers were conduct problems (37.3%, n = 259) and the least frequent were relationship problems with peers (21.9%, n = 152).

The association between maternal depression trajectory groups and children's mental health problems at 5 years showed no gender interaction effect in relation to any of the outcomes investigated.

Logistic regression analyses were conducted to examine differences in children's mental disorders by maternal depression trajectory group after controlling for several potential confounders (Table 4). Single mother, maternal skin colour, alcohol consumption during pregnancy, planned pregnancy, type of delivery, child's sex, and preterm birth were not included in any of the models due to not being associated with maternal trajectories. The magnitude of risk for mental disorders in all maternal depressive trajectories compared with the "low" group (reference category) did not substantially change after adjusting for maternal and child characteristics. Children of mothers in the "increasing" and "high-chronic" trajectory groups showed the highest odds of having any SDQ difficulty, emotional problems, and hyperactivity/inattention symptoms. Offspring of mothers in the "increasing" group had the highest odds of experiencing conduct problems at age 5. Maternal depression trajectories were not associated with peer problems at age 5.

**Table 4:** Crude and adjusted analyses for mental health problems at 5 years according to trajectories of maternal depressive symptoms (children of mothers in the "low" depression trajectory = reference).

Child's mental health problems	Models	Maternal depression trajectory groups				p-value
		Group 1 "Low"	Group 2 "Increasing"	Group 3 "Decreasing"	Group 4 "High/chronic"	
		OR (95%CI)	OR (95%CI)	OR (95%CI)	OR (95%CI)	
Any SDQ difficulty	Model 1 = crude analysis	1.00 (ref.)	2.97 (1.90–4.63)	1.49 (0.96–2.32)	3.87 (1.62–9.29)	< 0.001
	Model 2 = Model 1 + maternal characteristics^a^	1.00 (ref.)	3.24 (2.01–5.23)	1.33 (0.83–2.13)	2.87 (1.10–7.52)	< 0.001
	Model 3 = Model 2 + child's variables^b^	1.00 (ref.)	3.23 (2.00–5.22)	1.27 (0.78–2.04)	2.87 (1.09–7.57)	< 0.001
Emotional symptoms	Model 1 = crude analysis	1.00 (ref.)	2.40 (1.55–3.74)	1.25 (0.81–1.92)	3.23 (1.33–7.86)	< 0.001
	Model 2 = Model 1 + maternal characteristics^a^	1.00 (ref.)	2.54 (1.59–4.05)	1.18 (0.75–1.85)	2.58 (0.98–6.76)	0.001
	Model 3 = Model 2 + child's variables^b^	1.00 (ref.)	2.49 (1.56–3.98)	1.12 (0.71–1.77)	2.64 (1.00–6.96)	0.001
Hyperactivity/inattention symptoms	Model 1 = crude analysis	1.00 (ref.)	1.63 (1.05–2.56)	1.19 (0.76–1.85)	2.78 (1.17–6.58)	0.028
	Model 2 = Model 1 + maternal characteristics^a^	1.00 (ref.)	1.80 (1.12–2.89)	1.08 (0.68–1.73)	2.26 (0.87–5.87)	0.047
	Model 3 = Model 2 + child's variables^b^	1.00 (ref.)	1.81 (1.13–2.91)	1.09 (0.68–1.75)	2.20 (0.84–5.75)	0.049
Conduct problems	Model 1 = crude analysis	1.00 (ref.)	2.32 (1.49–3.59)	1.53 (1.00–2.34)	2.09 (0.89. 4.94)	0.001
	Model 2 = Model 1 + maternal characteristics^a^	1.00 (ref.)	2.39 (1.49–3.84)	1.42 (0.90–2.23)	1.25 (0.47–3.36)	0.003
	Model 3 = Model 2 + child's variables^b^	1.00 (ref.)	2.41 (1.50–3.87)	1.38 (0.87–2.20)	1.21 (0.45–3.29)	0.004
Peer problems	Model 1 = crude analysis	1.00 (ref.)	1.93 (1.20–3.12)	1.02 (0.61–1.72)	1.52 (0.58–3.98)	0.059
	Model 2 = Model 1 + maternal characteristics^a^	1.00 (ref.)	1.84 (1.09–3.08)	0.85 (0.49–1.48)	1.83 (0.64–5.17)	0.060
	Model 3 = Model 2 + child's variables^b^	1.00 (ref.)	1.84 (1.09–3.08)	0.82 (0.47–1.44)	1.76 (0.61–5.03)	0.056

OR: odds ratio; 95%CI: 95% confidence interval; ref.: reference.

a Wealth index, schooling, maternal age, smoking during pregnancy, planned pregnancy, parity, and number of prenatal care visits.

b Low birthweight and preterm birth.

## DISCUSSION

We identified four trajectories of maternal depressive symptoms from 3 months to 5 years after childbirth: a "low" trajectory (67.1%), an "increasing" trajectory (11.5%), a "decreasing" trajectory (17.4%), and a "high-chronic" trajectory (4.0%). Poverty, low education, advanced maternal age, multiparity, smoking during pregnancy, and fewer antenatal consultations were characteristics more frequently observed among mothers who belonged to the "high/chronic" trajectory. Offspring of mothers belonging to the "increasing" and "high-chronic" trajectory groups showed the highest odds of any SDQ difficulty, emotional, and hyperactivity/inattention symptoms at age 5. Maternal and child variables included in the adjusted analyses did not substantially explain these differences.

Maternal depression is not a homogeneous entity and has different clinical courses according to the severity, chronicity, and timing of mothers' depressive symptoms. Several studies have explored longitudinal analysis of maternal depressive symptoms using different methodologies, such as Group-Based Modelling, Latent Profile Analysis, and Growth Mixture Modeling^17^. Most studies identified between three and five trajectories^21–23^. While most women in the studies belonged to the "low or no depressive symptomatology" trajectories, around 5–12% of women were most impaired showing high and persistent depressive symptoms over time. Predictors of all patterns of depression varied in the different studies, and the most frequent were family socioeconomic position, maternal age, smoking during pregnancy, and partner and social support.

Our results are in line with previous studies carried out in high income countries such as in the Netherlands^24^, Australia^25^, France^26^, and Korea^27^, where children of mothers belonging to the "high symptoms" trajectories had more emotional and behavioural difficulties than those of mothers belonging to the "low/no symptoms" trajectory group. A population-birth cohort study in the south of Brazil had similar findings, with the odds of having any psychiatric disorder, internalizing and externalizing problems being nearly seven-times higher among 6-year-old children of mothers belonging to the "high-chronic" trajectory compared with the "low" group^23^. In Chile, Chae et al.^28^ evaluated 1,273 mothers from one to 14.6 years after childbirth and reported that exposure to maternal depression of any duration, severity, or time-period during childhood was associated with higher levels of externalizing and attention problems at both age 14.6 and 20.5 years and higher levels of internalizing problems at adulthood^28^.

This study has many strengths. First, the MINA-Brazil cohort was set up to better understand the effects of early environmental exposures on the growth and development of Amazonian children, and no previous research in this region looked at the impact of maternal depression on child mental health. Second, we used well validated instruments for assessing both maternal depressive symptomatology and offspring mental health. Third, we adjusted for several confounding variables. However, several limitations must also be considered. First, almost 40% of the original cohort, mainly women and their children in disadvantaged conditions were not included in this study. Even though losses to follow-up could have affected maternal depression and child mental health problems prevalences, the association between maternal depression trajectories and offspring mental health is less likely to be affected^29^. Second, we did not have other informants of the child's mental health problems and we had to rely on the maternal report alone. However, a previous study showed that depressed mothers could be as accurate as other informants regarding their children behaviour^30^. Finally, even though genetic factors or genotype-environment interactions could play a significant role in the genesis of children mental health problems, this information was unavailable in our study.

## CONCLUSION

This study provided evidence of different trajectories of maternal postnatal depressive symptomatology and their impact on children's mental health. We identified a small but highly important group of mothers with chronic and severe symptoms of depression throughout the first five years of the child life. These results are interesting since most mothers during the first years after birth are in contact with health professionals, mainly due to childcare consultations, when women at risk of persistent depressive symptomatology could be identified and treated. Our findings support the need to provide mental health evaluation and care for women and their children thus avoiding the long-term effects of maternal depression on offspring mental health and well-being.
