# Supplementary material for: Maternal depression and offspring mental health at age 5: MINA-Brazil cohort study
Source: Rev Saude Publica. 2024 Feb 1;57(Suppl 2):5s. doi: 10.11606/s1518-8787.2023057005560 (PMC10897964; doi:10.11606/s1518-8787.2023057005560)
Supplement: Translation

# Translation: Depressão materna e saúde mental infantil aos cinco anos de idade: Estudo de coorte MINA-Brasil

**Authors:** Alicia Matijasevich, Alexandre Faisal-Cury, Isabel Giacomini, Julia de Souza Rodrigues, Marcia C. Castro, Marly A. Cardoso

**Affiliations:** I São Paulo SP Brasil Universidade de São Paulo. Faculdade de Medicina. Departamento de Medicina Preventiva. São Paulo, SP, Brasil; II São Paulo SP Brasil Universidade de São Paulo. Faculdade de Saúde Pública. Programa de Pós-Graduação em Nutrição em Saúde Pública. São Paulo, SP, Brasil; III São Paulo SP Brasil Universidade de São Paulo. Faculdade de Medicina. Programa de Pós-Graduação em Saúde Coletiva. São Paulo, SP, Brasil; IV Boston MA United States of America Harvard T.H. Chan School of Public Health. Department of Global Health and Population. Boston, MA, United States of America; V São Paulo SP Brasil Universidade de São Paulo. Faculdade de Saúde Pública. Departamento de Nutrição. São Paulo, SP, Brasil

**Keywords:** Saúde Mental, Depressão Materna, Estudos de Coortes

## Abstract

**OBJETIVO:**

Identificar padrões longitudinais de depressão materna entre três meses e cinco anos após o nascimento de seus filhos, analisar variáveis preditoras dessas trajetórias e avaliar se trajetórias distintas de depressão predizem problemas de saúde mental infantil aos cinco anos de idade.

**MÉTODOS:**

Utilizou-se dados do estudo sobre saúde e nutrição materno infantil no Acre (MINA-Brasil), uma coorte de nascimentos de base populacional na Amazônia ocidental brasileira. Os sintomas depressivos maternos foram avaliados pela Escala de Depressão Pós-parto de Edimburgo (EPDS) aos 3 e 6–8 meses e 1 e 2 anos após o parto. Problemas de saúde mental em crianças com cinco anos de idade foram avaliados pelo Questionário de Capacidades e Dificuldades (SDQ–* Strengths and Difficulties Questionnaire*), respondido pelos pais. As trajetórias de depressão materna foram calculadas usando uma abordagem de modelagem baseada em grupos.

**RESULTADOS:**

Foram identificadas quatro trajetórias de sintomas depressivos maternos: "baixa" (67,1%), "crescente" (11,5%), "decrescente" (17,4%) e "alta-crônica" (4,0%). As mulheres na trajetória "alta/crônica" eram mais pobres, menos escolarizadas, mais velhas e multíparas e relataram tabagismo com maior frequência e menor número de consultas de pré-natal durante a gestação do que as demais. Nas análises ajustadas, a razão de chances de qualquer transtorno do SDQ foi 3,23 (IC95%:2,00–5,22) e 2,87 (IC95%: 1,09–7,57) vezes maior entre os filhos de mães nos grupos de trajetória "crescente" e "alta-crônica", respectivamente, do que de mães do grupo de sintomas depressivos "baixos". As características maternas e infantis incluídas nas análises multivariadas foram incapazes de explicar essas diferenças.

**CONCLUSÕES:**

Identificou-se piores desfechos de saúde mental para filhos de mães atribuídas às trajetórias "crônica/grave" e "crescente" de sintomas depressivos. Iniciativas de prevenção e tratamento para evitar os efeitos adversos a curto, médio e longo prazo da depressão materna sobre o desenvolvimento de seus filhos devem se concentrar principalmente nas mulheres nesses grupos.

## INTRODUÇÃO

A depressão é um dos mais importantes problemas de saúde pública em todo o mundo, afetando principalmente as mulheres^1^. A depressão perinatal, um problema de saúde pública global e mais frequente entre mulheres de países de baixa e média renda, engloba transtornos depressivos maiores e menores durante a gravidez ou até 12 meses após o parto^2^. A depressão pós-parto (DPP) pode tanto ocorrer durante o primeiro ano após o parto quanto continuar por vários anos. Uma revisão recente da literatura com 565 estudos de 80 países ou regiões diferentes encontrou uma prevalência de DPP de 17,2% (IC95%: 16,0–18,5), com valores mais elevados entre países de renda média e baixa^3^.

A depressão materna ocorre frequentemente em um contexto de problemas socioeconômicos, eventos estressantes, dificuldades conjugais, pouco suporte social e de mães solteiras. Entre as mulheres vulneráveis, complicações durante a gravidez e o parto e problemas de saúde infantil também podem desencadear sintomas depressivos^4^. Embora os casos graves de DPP sejam facilmente detectáveis, os casos moderados são frequentemente confundidos com cansaço, distúrbios do sono ou passam completamente despercebidos por familiares e profissionais de saúde^5^. A maioria dos sintomas depressivos após o parto é autolimitada, desaparecendo em poucos meses após seu início^6^. No entanto, os sintomas depressivos maternos podem se tornar crônicos ou recorrentes já que um histórico de depressão e de depressão perinatal ao longo da vida são importantes fatores de risco para DPP^7^.

Além dos prejuízos à qualidade de vida da mulher e seu impacto negativo nas relações familiares, a depressão materna também tem consequências negativas no desenvolvimento infantil. A exposição à DPP leva a desfechos negativos em múltiplos domínios da saúde infantil, desde maiores taxas de prematuridade, baixo peso ao nascer e desnutrição e prejuízo ao desenvolvimento linguístico, cognitivo e motor^8^. A DPP também está associada a um maior risco de dificuldades comportamentais infantis e transtornos internalizantes e externalizantes persistentes com efeitos que vão muito além da infância^9^.

Evidências clínicas têm mostrado padrões e preditores heterogêneos na sintomatologia depressiva materna ao longo do tempo. Uma revisão sistemática recente identificou nove estudos (cuja maioria fora conduzida em países de alta renda) que modelaram trajetórias de depressão materna e estudaram o impacto dessas trajetórias em transtornos psiquiátricos infantis^10^. Este estudo realizado na Amazônia tem três objetivos principais: 1) identificar trajetórias de depressão materna entre três meses e cinco anos após o nascimento da criança, 2) examinar variáveis preditoras dessas trajetórias e 3) avaliar se tais trajetórias predizem problemas de saúde mental infantil aos cinco anos de idade.

## MÉTODOS

### Participantes

O estudo de saúde e nutrição materno infantil no Acre (MINA-Brasil) é uma coorte de nascimentos de base populacional com crianças nascidas entre julho de 2015 e junho de 2016 na cidade de Cruzeiro do Sul, estado do Acre, Brasil. O município fica na Amazônia Ocidental (na região Norte do país) e tem uma população estimada em 88.376 pessoas em 2019. Em 2010, Cruzeiro do Sul tinha um índice de desenvolvimento humano igual a 0,664 (a média brasileira é de 0,727). A equipe de campo visitou diariamente a única maternidade da cidade e convidou todas as mulheres que deram à luz no hospital a participar do estudo. As mães foram entrevistadas logo após o parto por um questionário estruturado. Foram obtidas informações sobre características demográficas, socioeconômicas, comportamentais, biológicas, história reprodutiva e utilização de serviços de saúde. Características dos recém-nascidos como sexo, idade gestacional, peso e comprimento ao nascer foram obtidas de prontuários hospitalares. O seguimento basal incluiu 1.246 crianças nascidas de mães residentes na zona urbana da cidade. Apenas partos únicos foram incluídos no estudo. Mães e crianças residentes na área rural remota da cidade foram consideradas inelegíveis para acompanhamento. Após o nascimento, a visita de seguimento de três meses foi realizada por entrevista telefônica. As avaliações aos seis a oito meses e um, dois e cinco anos após o parto foram agendadas por telefone e realizadas em uma unidade básica de saúde do município. Informações sobre diversas características da família, mãe e criança foram coletadas durante os acompanhamentos e até três tentativas de reagendamento foram realizadas em caso de não comparecimento. Mais detalhes da metodologia foram descritos anteriormente^11^.

### Mensurações

**Sintomas depressivos maternos:** as avaliações dos sintomas depressivos maternos aos 3 e 6–8 meses e 1 e 2 anos após o parto foram feitas usando a Escala de Depressão Pós-natal de Edimburgo (EPDS).^12^ Esta escala autoaplicável contém 10 itens. Cada item possui quatro possibilidades de respostas que variam de 0 a 3, produzindo um escore mínimo total de 0 e máximo de 30. A escala indica a intensidade dos sintomas depressivos nos últimos sete dias. Uma versão brasileira do questionário, validada previamente no Brasil^13^, foi utilizada. O estudo de validação mostrou que uma pontuação de corte ≥ 10 identificou mulheres em risco de depressão menor com sensibilidade de 82,6% (75,3–89,9%) e especificidade de 65,4% (59,8–71,1%). A EPDS também foi validada para uso na população adulta fora do período pós-parto^14^. No estudo MINA-Brasil, os escores maternos na EPDS se correlacionaram moderadamente ao longo do tempo (de 0,41 a 0,67).

**Avaliação da saúde mental da criança:** no acompanhamento aos cinco anos, as mães foram entrevistadas por trabalhadores de campo treinados usando o Questionário de Capacidades e Dificuldades (SDQ–* Strengths and Difficulties Questionnaire*): um breve questionário de triagem comportamental para crianças de dois a 17 anos de idade. O SDQ foi validado para uso no Brasil por Fleitlich-Bilyk e Goodman^15^. O instrumento é composto por 25 questões divididas em cinco subescalas com cinco itens cada: sintomas emocionais, problemas de conduta, hiperatividade, problemas de relacionamento com colegas e comportamento pró-social. Cada questão é avaliada por uma escala de três pontos (falso = 0, mais ou menos verdadeiro = 1 e verdadeiro = 2). Foram utilizados os escores totais do SDQ e os das subescalas sintomas emocionais, conduta, hiperatividade e problemas de relacionamento com colegas. O escore total de dificuldade (qualquer dificuldade do SDQ) foi calculado somando-se os resultados das subescalas (exceto comportamento pró-social). O escore resultante variou de 0 a 40 e pode ser estratificado em três categorias: normal (0–13), limítrofe (14–16) e anormal (17–40). Os escores das subescalas variaram de 0 a 10 e foram categorizados em normal, limítrofe ou anormal de acordo com os pontos de corte sugeridos no site do SDQ^16^. Utilizou-se uma classificação dicotômica dos escores do SDQ em que os indivíduos nas categorias limítrofe e anormal foram comparados com os do grupo normal.

**Covariáveis:** as informações sobre as variáveis maternas e infantis foram coletadas na entrevista perinatal. Um índice de riqueza foi estimado pela análise de componentes principais, baseando-se nas posses domésticas de bens de consumo e nas características de infraestrutura e habitação e categorizado em quintis, sendo o 1º e o 5º os grupos mais pobres e ricos, respectivamente. A escolaridade materna no momento do parto foi registrada como anos completos e categorizada em ≤ 9, 10–12 e > 12 anos de educação formal. A idade materna foi registrada em anos completos e categorizada em < 19, 20–35 e > 35 anos. Mulheres solteiras, viúvas, divorciadas ou que moravam em um domicílio diferente do(a) seu(sua) parceiro(a) foram classificadas como mães solteiras. A cor da pele materna foi autorreferida e categorizada em branca ou preta/parda. O tabagismo materno durante a gestação foi avaliado retrospectivamente ao nascimento por autorrelato. As mulheres que fumavam pelo menos um cigarro por dia em qualquer trimestre da gestação foram definidas como tabagistas regulares. A ingestão de qualquer quantidade de álcool durante qualquer trimestre da gestação foi considerada como consumo de álcool durante a gestação. As mulheres foram questionadas sobre o planejamento de sua gravidez (sim/não). Paridade foi definida como o número de gestações viáveis anteriores e categorizada em 1 (primiparidade) e ≥ 2. O número de consultas de pré-natal foi categorizado em < 6 e ≥ 6. O tipo de parto foi classificado como vaginal ou cesariana.

As variáveis infantis foram registradas ao nascimento. O peso ao nascer foi aferido pela equipe do hospital. Baixo peso ao nascer foi definido como abaixo de 2.500g. A idade gestacional foi baseada na última menstruação, ultrassonografia e maturidade clínica do recém-nascido. Os nascimentos antes da 37ª semana de gestação foram classificados como prematuros.

### Plano de Análise

Trajetórias de sintomas depressivos baseadas em escores de EPDS entre três meses e cinco anos de seguimento foram criadas por uma abordagem de modelagem semiparamétrica baseada em grupos, uma forma especializada de modelagem de mistura finita^17^. O método é projetado para identificar (em vez de supor) grupos ou *clusters* de indivíduos que seguem trajetórias de desenvolvimento semelhantes. Uma função polinomial é usada para modelar a relação entre um atributo (isto é, sintomatologia de depressão materna) e idade ou tempo^17,18^. Os modelos foram estimados com o procedimento "traj" do Stata^19^. Foram incluídas 1.141 mulheres com dados válidos da EPDS em pelo menos uma consulta de seguimento. A proporção de mulheres que completaram a EPDS em todos os acompanhamentos foi de 29,4%. No geral, 71,4% completaram a EPDS pelo menos três vezes; 16,2%, duas, e 12,4%, apenas uma vez. Indivíduos com informação ignorada foram mantidos no modelo já que a modelagem de trajetória baseada em grupo manipula dados faltantes usando estimativas de máxima verossimilhança^17^. As mulheres que não completaram a EPDS (n = 105) foram excluídas das análises.

Para estimar as trajetórias de depressão materna, um modelo normal censurado (CNORM) foi ajustado aos dados. O número e a forma das trajetórias foram escolhidos com base no melhor ajuste do critério máximo de informação bayesiana do modelo, na interpretabilidade das trajetórias obtidas e nos escores de probabilidade posteriores para cada grupo de trajetória (a probabilidade de o indivíduo pertencer a cada um dos grupos de trajetória). De acordo com Nagin^17^, um escore de probabilidade médio deve exceder 0,70 para todos os grupos.

Em uma segunda etapa das análises, examinou-se a contribuição das variáveis preditoras (características maternas e da criança) sobre o grupo da trajetória da depressão materna. Como as variáveis foram categóricas, utilizou-se o teste qui-quadrado para comparar os grupos de acordo com as características maternas e da criança.

Na terceira etapa, avaliamos se a adesão ao grupo de trajetória de depressão materna prediz os problemas de saúde mental da criança aos cinco anos de idade. Obteve-se informações válidas sobre o SDQ para 694 crianças. A análise de regressão logística multivariada foi utilizada para estimar a associação entre qualquer dificuldade do SDQ, sintomas emocionais, problemas de conduta, hiperatividade, transtornos de problemas de relacionamento com colegas (desfechos binários) e trajetórias de sintomas depressivos maternos (exposição), ajustando-os para potenciais fatores de confusão em modelos separados. Potenciais variáveis de confusão foram incluídas na análise ajustada por seleção *backward*. Cada desfecho continha três modelos: resultados não ajustados (modelo 1), resultados ajustados para características maternas (modelo 2) e resultados ajustados para variáveis do segundo modelo e características da criança (modelo 3). Se o nível de significância fosse inferior a 0,20^20^, a variável permanecia no modelo como um confundidor em potencial para o próximo nível. Termos de interação entre trajetórias maternas de sintomas depressivos e sexo da criança foram testados, mas ignorados pelo modelo dada sua insignificância estatística. Todas as análises foram realizadas com o software Stata versão 17.0 (StataCorp LP, College Station, Tex).

### Ética

O protocolo de estudo e todos os acompanhamentos do estudo de coorte MINA-Brasil foram aprovados pelo Comitê de Ética da Universidade de São Paulo. Um termo de consentimento livre e esclarecido assinado foi utilizado em cada seguimento após as mães serem informadas sobre os objetivos deste estudo. A confidencialidade dos dados e a participação voluntária foram garantidas durante todo o processo, assim como a opção de desistência do estudo a qualquer momento sem a necessidade de se apresentar um motivo. As mães identificadas com problemas graves de saúde mental foram encaminhadas para as unidades de saúde locais apropriadas.

## RESULTADOS

### Análises de Atrito

Das 1.246 participantes, 1.141 (91,6%) tinham dados válidos na EPDS advindos de pelo menos um seguimento, sendo que destas, 694 responderam ao questionário SDQ (56% da coorte original). Entre os membros excluídos das análises das trajetórias de depressão materna e das análises da associação entre trajetórias de depressão materna e problemas de saúde mental infantil, as mulheres pertencentes às famílias mais pobres e com menor escolaridade, que eram adolescentes, consumiram álcool durante a gestação e tiveram o menor número de consultas de pré-natal foram super-representadas (Tabela 1).

**Tabela 1:** Características basais das mães e crianças incluídas excluídas neste estudo.

Variáveis		Análise das trajetórias da depressão materna			Análise da associação entre trajetórias de depressão materna e problemas de saúde mental infantil		
		Incluídos	Excluídos	valor-p	Incluídos	Excluídos	Valor-p^a^
		n = 1.141	n = 105		n = 694	n = 552	
Índice de riqueza (quintis) (%)				0.005			< 0.001
	1º (mais pobre)	19,0	29,3		13,0	28,6	
	2º	19,4	28,3		16,4	24,7	
	3º	20,4	14,1		22,0	17,2	
	4º	20,3	18,5		23,2	16,3	
	5º (mais rico)	20,9	9,8		25,4	13,2	
Escolaridade (anos) (%)				0.004			<0.001
	≤ 9	34,2	51,1		26,2	47,3	
	10–12	47,8	38,0		51,2	41,8	
	> 12	18,0	10,9		22,6	10,9	
Idade materna (anos) (%)				0.020			< 0.001
	< 19	17,9	28,3		16,0	22,1	
	20–35	72,4	66,7		72,1	71,8	
	> 35	9,7	5,0		11,9	6,1	
Mãe solteira (%)				0.495			0.329
	Não	77,3	80,4		78,6	76,3	
	Sim	22,7	19,6		21,4	23,7	
Cor da pele materna (%)				0.009			0.799
	Branca	12,0	18,5		12,4	12,6	
	Preta/parda	82,1	69,6		81,6	80,5	
	Amarela/Indígena	5,9	11,9		6,0	6,9	
Tabagismo na gestação (%)				0.187			0.028
	Não	95,4	92,4		96,4	93,7	
	Sim	4,6	7,6		3,6	6,3	
Consumo de álcool durante a gestação (%)				0.001			0.002
	Não	83,0	69,6		85,0	78,0	
	Sim	17,0	30,4		15,0	22,0	
Gravidez planejada (%)				0.878			0.182
	Não	60,0	60,9		58,4	62,3	
	Sim	40,0	39,1		41,6	37,7	
Paridade (%)				0.578			0.613
	1 (Primiparidade)	41,6	44,6		42,4	41,0	
	≥ 2	58,4	55,4		57,6	59,0	
Número de consultas de pré-natal (%)				< 0.001			< 0.001
	< 6	22,0	43,9		16,3	33,1	
	≥ 6	78,0	56,1		83,7	66,9	
Tipo de parto (%)				0.582			0.041
	Vaginal	53,7	56,6		51,3	57,2	
	Cesariana	46,3	43,4		48,7	42,8	
Sexo da criança (%)				0.527			0.337
	Masculino	49,8	53,5		50,7	48,0	
	Feminino	50,2	46,5		49,3	52,0	
Baixo peso ao nascer (< 2.500g) (%)				0.213			0.382
	Não	93,2	89,9		93,5	92,3	
	Sim	6,8	10,1		6,5	7,7	
Prematuridade (< 37 semanas) (%)				0.013			0.280
	Não	92,1	84,8		92,3	90,6	
	Sim	7,9	15,2		7,7	9,4	

a Teste qui-quadrado.

### Descrição da amostra

Cerca de metade das mulheres tinha de 10 a 12 anos de educação formal, 70% tinham entre 20 e 35 anos, uma em cada cinco eram mães solteiras, a maioria se identificaram como preta ou parda, não-fumantes, quem não consumiram álcool durante a gestação, realizaram seis ou mais consultas de pré-natal, e cerca de 40% eram primíparas e não planejavam sua gravidez. A via de parto mais frequente foi a vaginal e cerca de 7 e 8% das crianças tinham baixo peso ao nascer e eram prematuras, respectivamente.

### Identificação de Trajetórias

Modelamos as trajetórias dos escores maternos da EPDS dos três meses aos cinco anos de idade de seus filhos. As análises especificaram modelos de três, quatro, cinco e seis grupos. O critério máximo de informação bayesiana melhorou proporcionalmente à adição de grupos, mas a melhora observada do modelo de quatro para cinco e seis grupos foi muito baixa e o modelo de quatro grupos emergiu como o modelo de melhor ajuste e parcimônia. Em relação às estimativas dos parâmetros para o modelo de quatro grupos, a Tabela 2 mostra que o termo constante diferiu de zero para os quatro grupos. No total, duas trajetórias foram melhor representadas por um termo cúbico, uma trajetória foi linear e a outra quadrática (Figura). O grupo 1 ("baixo", n = 765, 67,1%) apresentou escores da EPDS abaixo de cinco em todos os momentos, sugerindo baixa sintomatologia depressiva. O grupo 2 ("crescente", n = 131, 11,5%) apresentou escores da EPDS abaixo de 10 nos primeiros 24 meses pós-parto, com aumento acentuado entre os 24 meses e os cinco anos de seguimento. O grupo 3 ("decrescente", n = 199, 17,4%) apresentou diminuição consistente dos sintomas depressivos durante o período estudado. Finalmente, o grupo 4 ("alto/crônico", n = 46, 4,0%) representa as mulheres que apresentaram escores muito altos na EPDS durante todo o período do estudo. Para os quatro grupos, a média de probabilidade a posteriori (MPP) esteve acima do limite inferior recomendado para atribuição, 0,7 (MPP de 0,89, 0,74, 0,73 e 0,87 do grupo 1 ao 4, respectivamente).

**Figure:** Trajetórias dos sintomas de depressão materna medidas pela Escala de Depressão Pós-parto de Edimburgo (EPDS) de acordo com a idade da criança.

**Tabela 2:** Probabilidades a posteriori médias (MPP) e descritivas para as trajetórias (n = 1.141).

Trajetórias dos sintomas depressivos	n (%)	MPP (DP)	Parâmetros	Estimativas de parâmetros		
				B	(SE)	Valor-p
1 Sintomas depressivos "baixos"	765 (67,1)	0,89 (0,15)	Intercepto	6,205	(0,312)	< 0,001
			Linear	−0,503	(0,071)	< 0,001
			Quadrático	0,022	(0,003)	< 0,001
			Cúbico	−0,000	(0,000)	< 0,001
2 Sintomas depressivos "crescentes"	131 (11,5)	0,74 (0,22)	Intercepto	9,474	(0,551)	< 0,001
			Linear	−0,225	(0,048)	< 0,001
			Quadrático	0,005	(0,001)	< 0,001
3 Sintomas depressivos "decrescentes"	199 (17,4)	0,73 (0,19)	Intercepto	11,861	(0,428)	< 0,001
			Linear	−0,112	(0,015)	< 0,001
4 Sintomas depressivos "altos/crônicos"	46 (4,0)	0,87 (0,17)	Intercepto	14,081	−1,336	< 0,001
			Linear	0,784	(0,309)	0,011
			Quadrático	−0,034	(0,015)	0,027
			Cúbico	0,000	(0,000)	0,037

### Fatores Associados à Trajetória de Depressão Materna

As mulheres no grupo trajetória de depressão "alta/crônica" pertenciam às famílias mais pobres e eram mais frequentemente as menos escolarizadas, velhas e multíparas. Elas também relataram com mais frequência fumar durante a gestação e ter realizado menos consultas de pré-natal do que as demais. Em relação às características das crianças, o baixo peso ao nascer foi maior entre os filhos de mães na trajetória alta/crônica do que entre os filhos das demais mães (Tabela 3).

**Tabela 3:** Características maternas e infantis segundo grupos de trajetória de depressão materna.

Variáveis^a^		Grupos da trajetória da depressão materna				Valor-p^b^
		Grupo 1 "Baixa"	Grupo 2 "Crescente"	Grupo 3 "Decrescente"	Grupo 4 "Alta/crônica"	
		n (%)	n (%)	n (%)	n (%)	
Índice de riqueza (quintis)						< 0,001
	1º (mais pobre)	120 (16,3)	24 (19,0)	48 (24,6)	17 (40,5)	
	2º ao 5º	616 (83,7)	102 (81,0)	147 (75,4)	25 (59,5)	
Escolaridade (anos)						< 0.001
	≤ 9	218 (29,7)	49 (38,9)	83 (42,6)	25 (59,6)	
	10–12	353 (48,0)	62 (49,2)	96 (49,2)	14 (33,3)	
	> 12	164 (22,3)	15 (11,9)	16 (8,2)	3 (7,1)	
Idade materna (anos)						0.015
	< 19	139 (18,4)	21 (16,3)	39 (19,7)	2 (4,6)	
	20–35	551 (73,1)	91 (70,5)	141 (71,2)	32 (72,7)	
	> 35	64 (8,5)	17 (13,2)	18 (9,1)	10 (22,7)	
Mãe solteira						0.380
	Não	563 (76,5)	95 (75,4)	160 (82,0)	32 (76,2)	
	Sim	173 (23,5)	31 (24,6)	35 (18,0)	10 (23,8)	
Cor da pele materna						0.644
	Branca	86 (11,7)	16 (12,7)	21 (10,8)	9 (21,4)	
	Preta/parda	608 (82,6)	102 (81,0)	161 (82,5)	31 (73,8)	
	Amarela/Indígena	42 (5,7)	8 (6,3)	13 (6,7)	2 (4,8)	
Tabagismo durante a gravidez						< 0.001
	Não	713 (96,9)	119 (94,4)	183 (93,8)	34 (80,9)	
	Sim	23 (3,1)	7 (5,6)	12 (6,2)	9 (19,1)	
Consumo de álcool durante a gravidez						0.255
	Não	617 (83,8)	107 (84,9)	157 (80,5)	31 (73,8)	
	Sim	119 (16,2)	19 (15,1)	38 (19,5)	11 (26,2)	
Gravidez planejada						0.086
	Não	428 (58,1)	74 (58,7)	133 (68,2)	25 (59,5)	
	Sim	308 (41,9)	52 (41,3)	62 (31,8)	17 (40,5)	
Paridade						< 0.001
	1 (Primiparidade)	348 (47,3)	38 (30,2)	64 (32,8)	7 (16,7)	
	≥ 2	388 (52,7)	88 (69,8)	131 (67,2)	35 (83,3)	
Número de consultas de pré-natal						< 0.001
	< 6	146 (19,5)	26 (20,3)	54 (27,8)	19 (43,2)	
	≥ 6	604 (80,5)	102 (79,7)	140 (72,2)	25 (56,8)	
Tipo de parto						0.664
	Vaginal	397 (52,6)	70 (54,3)	114 (57,6)	23 (52,3)	
	Cesariana	357 (47,4)	59 (45,7)	84 (42,4)	21 (47,7)	
Sexo da criança						0.838
	Masculino	372 (49,3)	69 (53,5)	98 (49,5)	21 (47,7)	
	Feminino	382 (50,7)	60 (46,5)	100 (50,5)	23 (52,3)	
Baixo peso ao nascer (< 2.500g)						0.003
	Não	706 (93,8)	120 (93,0)	187 (94,4)	35 (79,5)	
	Sim	47 (6,2)	9 (7,0)	11 (5,6)	9 (20,5)	
Parto prematuro (< 37 semanas)						0.141
	Não	686 (91,5)	120 (93,0)	186 (95,4)	38 (86,4)	
	Sim	64 (8,5)	9 (7,0)	9 (4,6)	6 (13,6)	

a Os totais diferem devido a valores ausentes.

b Teste qui-quadrado.

### Problemas de saúde mental infantil aos cinco anos em função da trajetória de depressão materna

Em relação aos problemas de saúde mental das crianças, 33,1% (n = 230) apresentaram alguma dificuldade no SDQ aos cinco anos. Os sintomas emocionais e de hiperatividade totalizaram 39,9% (n = 277) e 33,1% (n = 230), respectivamente. Os transtornos mais frequentemente relatados pelas mães foram problemas de conduta (37,3%, n = 259) e os menos frequentes foram problemas de relacionamento com os colegas (21,9%, n = 152).

A associação entre os grupos da trajetória da depressão materna e os problemas de saúde mental das crianças aos cinco anos não mostrou efeito da interação de gênero em relação a nenhum dos desfechos investigados.

Análises de regressão logística foram conduzidas para examinar diferenças nos transtornos mentais infantis por grupo da trajetória de depressão materna após o controle de vários potenciais fatores de confusão (Tabela 4). A cor da pele materna, consumo de álcool durante a gestação, gravidez planejada, tipo de parto, sexo da criança, prematuridade e ser mãe solteira foram ignorados em todos os modelos por sua falta de associação às trajetórias maternas. A magnitude do risco para transtornos mentais em todas as trajetórias depressivas maternas em comparação com o grupo "baixo" (categoria de referência) não se alterou substancialmente após ajuste para características maternas e infantis. Filhos de mães nos grupos de trajetória "crescente" e "alta-crônica" apresentaram as maiores chances de ter alguma dificuldade no SDQ, problemas emocionais e sintomas de hiperatividade. Filhos de mães do grupo "crescente" apresentaram as maiores chances de apresentar problemas de conduta aos cinco anos de idade. As trajetórias de depressão materna não foram associadas a problemas com colegas aos cinco anos de idade.

**Tabela 4:** Análises brutas e ajustadas para problemas de saúde mental aos cinco anos segundo trajetórias de sintomas depressivos maternos (filhos de mães na trajetória de depressão "baixa" = referência).

Problemas de saúde mental infantil	Modelos	Grupos da trajetória da depressão materna				Valor-p
		Grupo 1 "Baixa"	Grupo 2 "Crescente"	Grupo 3 "Decrescente"	Grupo 4 "Alta/crônica"	
		OR (95%CI)	OR (95%CI)	OR (95%CI)	OR (95%CI)	
Qualquer dificuldade SDQ	Modelo 1 = análise bruta	1,00 (ref.)	2,97 (1,90–4,63)	1,49 (0,96–2,32)	3,87 (1,62–9,29)	< 0,001
	Modelo 2 = Modelo 1 + características maternas^a^	1,00 (ref.)	3,24 (2,01–5,23)	1,33 (0,83–2,13)	2,87 (1,10–7,52)	< 0,001
	Modelo 3 = Modelo 2 + variáveis infantis^b^	1,00 (ref.)	3,23 (2,00–5,22)	1,27 (0,78–2,04)	2,87 (1,09–7,57)	< 0,001
Sintomas emocionais	Modelo 1 = análise bruta	1,00 (ref.)	2,40 (1,55–3,74)	1,25 (0,81–1,92)	3,23 (1,33–7,86)	< 0,001
	Modelo 2 = Modelo 1 + características maternas^a^	1,00 (ref.)	2,54 (1,59–4,05)	1,18 (0,75–1,85)	2,58 (0,98–6,76)	0,001
	Modelo 3 = Modelo 2 + variáveis infantis^b^	1,00 (ref.)	2,49 (1,56–3,98)	1,12 (0,71–1,77)	2,64 (1,00–6,96)	0,001
Sintomas de hiperatividade	Modelo 1 = análise bruta	1,00 (ref.)	1,63 (1,05–2,56)	1,19 (0,76–1,85)	2,78 (1,17–6,58)	0,028
	Modelo 2 = Modelo 1 + características maternas^a^	1,00 (ref.)	1,80 (1,12–2,89)	1,08 (0,68–1,73)	2,26 (0,87–5,87)	0,047
	Modelo 3 = Modelo 2 + variáveis infantis^b^	1,00 (ref.)	1,81 (1,13–2,91)	1,09 (0,68–1,75)	2,20 (0,84–5,75)	0,049
Problemas de conduta	Modelo 1 = análise bruta	1,00 (ref.)	2,32 (1,49–3,59)	1,53 (1,00–2,34)	2,09 (0,89, 4,94)	0,001
	Modelo 2 = Modelo 1 + características maternas^a^	1,00 (ref.)	2,39 (1,49–3,84)	1,42 (0,90–2,23)	1,25 (0,47–3,36)	0,003
	Modelo 3 = Modelo 2 + variáveis infantis^b^	1,00 (ref.)	2,41 (1,50–3,87)	1,38 (0,87–2,20)	1,21 (0,45–3,29)	0,004
Problemas com colegas	Modelo 1 = análise bruta	1,00 (ref.)	1,93 (1,20–3,12)	1,02 (0,61–1,72)	1,52 (0,58–3,98)	0,059
	Modelo 2 = Modelo 1 + características maternas^a^	1,00 (ref.)	1,84 (1,09–3,08)	0,85 (0,49–1,48)	1,83 (0,64–5,17)	0,060
	Modelo 3 = Modelo 2 + variáveis infantis^b^	1,00 (ref.)	1,84 (1,09–3,08)	0,82 (0,47–1,44)	1,76 (0,61–5,03)	0,056

OR: razão de chances; IC95%: intervalo de confiança de 95%; Ref.: Referência.

a Índice de riqueza, escolaridade, idade materna, tabagismo na gestação, gestação planejada, paridade e número de consultas de pré-natal.

b Baixo peso ao nascer e prematuridade.

## DISCUSSÃO

Sintomas depressivos maternos de três meses a cinco anos após o parto mostraram quatro trajetórias: "baixa" (67,1%), "crescente" (11,5%), "decrescente" (17,4%) e "alta-crônica" (4,0%). Pobreza, baixa escolaridade, maior idade materna, multiparidade, tabagismo durante a gestação e menor número de consultas de pré-natal foram características mais frequentes entre as mães que pertenciam à trajetória "alta/crônica". Filhos de mães nos grupos de trajetória "crescente" e "alta-crônica" apresentaram as maiores chances de qualquer SDQ de dificuldade, emocional e sintomas de hiperatividade aos cinco anos de idade. As variáveis maternas e infantis incluídas nas análises ajustadas foram incapazes de explicar substancialmente essas diferenças.

A depressão materna é uma entidade heterogênea e tem vários cursos clínicos de acordo com a gravidade, cronicidade e tempo dos sintomas depressivos maternos. Vários estudos têm explorado a análise longitudinal de sintomas depressivos maternos usando diferentes metodologias, tais como modelagem baseada em grupo, análise de perfil latente e modelagem mistos de crescimento^17^. A maioria dos estudos identificou entre três e cinco trajetórias^21–23^. Embora a maioria das mulheres pertencesse às trajetórias de "sintomatologia depressiva baixa ou nenhuma" nesses estudos, cerca de 5-12% delas mostraram maior prejuízo, com sintomas depressivos altos e persistentes ao longo do tempo. Os preditores de todos os padrões de depressão variaram nestes estudos, sendo mais frequentes posição socioeconômica familiar, idade materna, tabagismo na gestação, presença de um companheiro e apoio social.

Nossos resultados estão de acordo com estudos realizados em países de alta renda, como Holanda^24^, Austrália^25^, França^26^, e Coreia^27^, onde filhos de mães pertencentes às trajetórias de "sintomas altos" apresentaram mais dificuldades emocionais e comportamentais do que os de mães no grupo de trajetória "baixo/nenhum sintoma". Um estudo de coorte de nascimentos populacionais realizado no sul do Brasil apresentou achados semelhantes, sendo a chance de algum transtorno psiquiátrico ou problema internalizante e externalizante quase sete vezes maior entre os filhos com seis anos de idade de mães pertencentes à trajetória "alta-crônica" do que aqueles do grupo "baixo". No Chile, Chae et al. avaliaram 1.273 mães entre um e 14,6 anos após o parto e relataram que a exposição à depressão materna (a despeito de duração, gravidade ou período de tempo) durante a infância foi associada a maiores níveis de problemas externalizantes e de atenção tanto aos 14,6 quanto aos 20,5 anos e níveis mais altos de problemas internalizantes na idade adulta.

Este estudo tem pontos fortes. Primeiro, a coorte MINA-Brasil foi criada para investigar os efeitos das exposições ambientais precoces no crescimento e desenvolvimento de crianças amazônicas e nenhuma pesquisa anterior nessa região analisou o impacto da depressão materna na saúde mental infantil. Segundo, usamos instrumentos bem validados para avaliar a sintomatologia depressiva materna e a saúde mental infantil. Terceiro, ajustou-se os modelos para várias variáveis de confusão. No entanto, várias limitações também devem ser consideradas. Primeiro, houve perda de seguimento de quase 40% da coorte original, principalmente mulheres e seus filhos em condições socio-econômicas desfavorecidas não foi exclusão, foram perdas de seguimento. Embora as perdas de seguimento possam ter afetado as prevalências de depressão materna e problemas de saúde mental infantil, a associação entre trajetórias de depressão materna e saúde mental infantil é menos afeita a tal efeito^29^. Em segundo lugar, não tínhamos outros informantes dos problemas de saúde mental infantil e tivemos que nos basear apenas em relatos maternos. No entanto, um estudo mostrou que mães com depressão podem ser tão precisas quanto outros informantes em relação ao comportamento de seus filhos^30^. Finalmente, embora fatores genéticos ou interações genótipo-ambiente possam desempenhar um papel importante na gênese de problemas de saúde mental infantil, essa informação esteve indisponível neste estudo.

## CONCLUSÃO

Este estudo evidenciou diferentes trajetórias da sintomatologia depressiva pós-natal materna e seu impacto na saúde mental infantil. Identificamos um grupo pequeno, mas muito importante de mães com sintomas depressivos crônicos ou graves ao longo dos primeiros cinco anos de vida de seus filhos. Esses resultados são interessantes já que a maioria das mães está em contato com profissionais de saúde nos primeiros anos após o parto, principalmente devido às consultas de puericultura, nas quais mulheres em risco de sintomatologia depressiva persistente puderam ser identificadas e tratadas. Nossos achados apoiam a necessidade de fornecer avaliação e cuidados de saúde mental para mulheres e seus filhos, evitando assim os efeitos a longo prazo da depressão materna na saúde mental e bem-estar infantis.
